# Resource Recovery From Spent Lithium Iron Phosphate Batteries via Low‐Temperature Lithium Extraction

**DOI:** 10.1002/gch2.70173

**Published:** 2026-11-13

**Authors:** Asad Ali, Yasir Arafat, Sandra Pavón, Amir Razmjou, Muhammad Rizwan Azhar

**Affiliations:** ^1^ School of Engineering Edith Cowan University Joondalup Western Australia Australia; ^2^ Department of Chemical Engineering University of Gujrat Gujrat Punjab Pakistan; ^3^ Fraunhofer‐Institut für Keramische Technologien und Systeme IKTS Freiberg Germany; ^4^ Mineral Resource Recovery Centre Edith Cowan University Joondalup Western Australia Australia

**Keywords:** battery recycling, hydrometallurgy, leaching, lithium iron phosphate, mixed control mechanism, oxalic acid, recycling

## Abstract

Lithium iron phosphate (LFP) batteries dominate electric‐vehicle and stationary‐storage markets, yet their low intrinsic metal value makes lithium recovery from spent material economically challenging. A low‐temperature oxalic acid leaching process recovers lithium from industrial LFP black mass without external oxidants. Box–Behnken response surface methodology (RSM) is combined with kinetic modeling to optimize the process. Temperature (35°C–70°C), oxalic acid concentration (0.30–1.00 M), and solid‐to‐liquid (S/L) ratio (20–100 g L^−1^) are evaluated over 180 min. The S/L ratio dominates lithium extraction, whereas temperature and acid concentration exert weak effects; extraction is essentially complete within 60–90 min. Kinetic analysis indicates that the Avrami model best describes the leaching behavior, consistent with mixed diffusion‐ and reaction‐controlled dissolution. Lithium is released by proton‐driven lattice dissolution while iron precipitates as amorphous iron oxalate, forming a product layer that restricts mass transfer; the low apparent activation energy supports diffusion‐influenced control. Leaching is selective for lithium over iron and copper, but aluminum and phosphorus are co‐extracted and require downstream separation. Under optimized conditions (35°C, 0.71 M oxalic acid, 45.4 g L^−1^), lithium recovery reaches 96%, matching model predictions. Efficient recovery is achievable at low temperature without oxidizing agents, providing a practical basis for sustainable battery recycling.

## Graphical abstract

Selective lithium recovery from industrial LiFePO_4_ black mass is achieved using low‐temperature oxalic acid leaching without external oxidants. Response surface optimisation enables 96% lithium recovery at 35°C, while kinetic analysis reveals diffusion‐influenced dissolution and iron oxalate precipitation. The findings demonstrate a promising route toward energy‐efficient and selective lithium recovery from spent batteries.

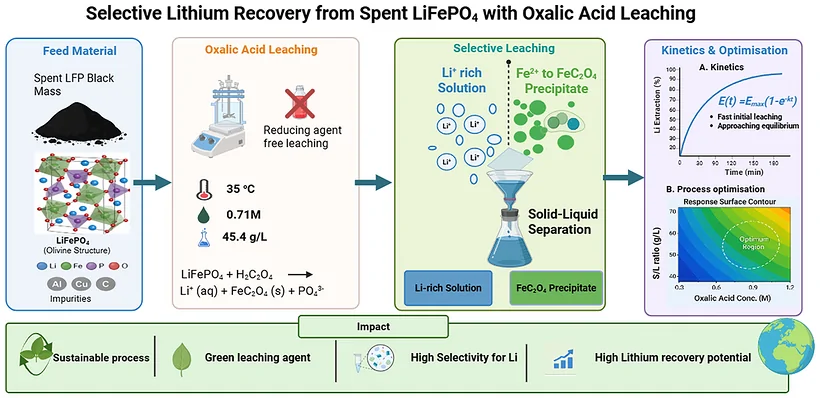

## 1 Introduction

To achieve global decarbonization, the transition toward electrification, particularly in transportation and energy storage, has led to a surge in demand for LIBs (Lithium‐ion batteries). LIBs are widely regarded as a clean and sustainable energy storage solution due to their high energy density and long cycle life. Consequently, their adoption has accelerated rapidly across multiple sectors [1]. It is projected that LIBs production will expand twentyfold by 2030 [2]. However, this exponential growth results in a substantial accumulation of End‐of‐Life (EoL) batteries [3]. These EoL batteries contain hazardous components and toxic heavy metals such as lithium hexafluorophosphate (LiPF_6_) [4], which pose serious health and environmental risks if not properly managed. At the same time, these batteries contain valuable metals, including Lithium (Li), Nickel (Ni), Cobalt (Co), Aluminum (Al), Iron (Fe), Phosphorus (P), and Copper (Cu) [5]. By 2035, global LIBs waste is projected to reach 6.76 million metric tons [6, 7], with LFP and Lithium manganese oxide (LMO) batteries expected to comprise nearly half (~45%) of the accumulated waste volume, highlighting the increasing importance of their recycling. Hence, developing efficient and sustainable recycling technologies to recover critical raw materials (CRMs) has become crucial for achieving high social, economic, and environmental benefits, aligning with circular economy objectives [7, 8, 9].

Among available LIBs in the market, LFP‐based batteries have grown rapidly due to their lower production cost, high safety, thermal stability, freedom from Co and Ni, and long lifespan [10, 11]. This has resulted in unprecedented production growth, particularly in China, where batteries based on this chemistry accounted for nearly 68% of the 778.2 GWh total power battery output in 2023. Market projections suggest continued expansion, with the global LFP battery sector expected to reach USD 67.39 billion by 2029 [12]. However, this rapid deployment inevitably leads to a large volume of EoL batteries, estimated at 313,300 tons worldwide by 2030. These batteries contain CRM, which are recoverable valuable resources, including Li and P, with an estimated economic value exceeding CNY 20 billion (≈ USD 2.8 billion). The accelerated commercialization of LFP batteries has highlighted a critical deficiency in sustainable and industrially viable recycling pathways. Inadequate recycling practices therefore cause environmental pollution and inefficient resource utilization [13, 14, 15]. Furthermore, most existing research focuses on pristine LFP materials, potentially limiting industrial feasibility due to insufficient consideration of black mass. LIBs obtained from recycling plants are typically present as black mass, a complex mixture containing cathode‐derived LiFePO_4_/FePO_4_ residues alongside anode materials such as graphite, current collector metals, and fluorinated binders, which can interfere with efficient leaching extraction.

Generally, the recycling of spent LFP batteries is classified into direct recycling and hydrometallurgy processes [16]. The former offers a structure‐conserving route that preserves the olivine phase of LFP cathode materials [17, 18, 19]. This process is particularly suitable for high‐purity active materials obtained through meticulous pretreatment, provided that extensive irreversible phase transformations have not occurred [20, 21]. However, its practical implementation remains limited to laboratory‐scale studies due to limitations in impurity removal, restricted material tolerance, and inconsistent electrochemical performance of regenerated products, as well as challenges in producing battery‐grade cathode materials at a large industrial scale [22, 23, 24]. By contrast, Hydrometallurgical recycling has emerged as the most adaptable and industrially relevant approach for processing EoL LFP batteries, owing to its high selectivity, relatively low energy consumption, and ability to process materials with complex compositions or those exhibiting irreversible lattice degradation [25]. Conventional hydrometallurgical pathways typically employ strong inorganic acids such as HCl, H_2_SO_4_, or H_3_PO_4_, often combined with reducing/oxidizing agents (e.g., H_2_O_2_) to facilitate metal dissolution [26]. Importantly, the redox function of H_2_O_2_ is cathode‐dependent. It commonly serves as a reductant during NMC leaching by facilitating the reduction of high‐valence transition metals to more soluble lower‐valence states, whereas in selective LFP leaching it can serve as an oxidant by promoting the oxidation of Fe^2+^ to Fe^3+^ and the concomitant release of Li^+^ from the LiFePO_4_ structure [27].

For instance, recent investigations demonstrate that the addition of oxidizing agents such as sodium persulfate (Na_2_S_2_0_8_) [26], sodium hypochlorite (NaClO) [28], peroxy‐monosulfate (PMS) [29], and hydrogen peroxide [30] enhances Li extraction. This is achieved by promoting oxidative dissolution while concurrently retaining iron as FePO_4_ in the solid fraction, enabling selective metal separation. Among these oxidants, H_2_O_2_ is preferred due to its environmentally benign oxidation product (H_2_O) [31]. Although effective, these systems introduce significant environmental and operational challenges, including equipment corrosion, toxic effluent generation, and emissions of poisonous gases such as Cl_2_, SO_x_, and NO_X_ [32]. Consequently, the development of greener hydrometallurgical strategies has become a critical research priority for advancing sustainable battery recycling technologies [33]. As a result, organic acids are increasingly being explored as sustainable alternatives to conventional inorganic acids in leaching steps [31].

Organic acids have emerged as an environmentally friendly alternative to conventional inorganic leaching agents for next‐generation LIBs recycling [34]. Owing to their strong chelating capability, organic acids exhibit high selectivity and can effectively leach valuable metals while minimizing secondary pollution. Several organic acids, including citric [35], ascorbic [36], tartaric [37], acetic [38], malic [39], formic [40], lactic [41], and oxalic acids [42, 43, 44], have demonstrated promising metal recovery efficiencies from spent LIBs. However, most studies have focused on high‐value ternary cathodes (NMC and LCO) [45], while systematic investigations on LFP‐based systems, especially using industrial black mass, remain limited.

Li, Luo, Zhang, Wang, Zhang, Wang, Yan, Hou, Wang, Zhang, Liu, Lei and Mu [46] demonstrated that a combined 0.33 M oxalic acid and 0.65 M phosphoric acid system enabled efficient recovery of Li (97.72%) and Fe (98.24%) at 70°C from EoL LFP‐based black mass through synergistic lattice disruption and iron chelation. Similarly, Chai, Yu, Shen, Li, Lin, Cai and Yuan [44] investigated low‐concentration oxalic acid (0.16–0.48 M) for two‐stage selective leaching, achieving ~99% Li extraction while limiting iron dissolution to only 2.4%. Furthermore, Ali, Iqbal, Noor and Zaman [47] developed an organic‐based leaching system using succinic acid and H_2_O_2_, achieving 98.7% Li extraction under optimized conditions (1.5 M acid, 4 Vol% H_2_O_2_, 70°C, 40 min), while limiting Fe and P dissolution to below 7%, thereby preserving FePO_4_ in the solid residue.

Previous studies on LFP leaching optimization achieved high lithium extraction efficiencies but relied on one factor at a time (such as optimization, extreme operating conditions, or the use of additional oxidants). Moreover, these studies typically lack detailed kinetic or mechanistic analysis, which is essential for understanding rate‐controlling steps and enabling process scale‐up [48]. Furthermore, the feasibility of energy‐efficient room‐temperature processing remains largely unexplored. In addition, the influence of impurities such as aluminum and copper present in industrial black mass on leaching selectivity and downstream processing remains insufficiently understood. Therefore, a critical knowledge gap exists in the development of an integrated framework that combines process optimization, kinetic modelling, and mechanistic understanding for the low‐temperature leaching of lithium from industrial LFP black mass.

To address this gap, this study investigates a low‐temperature oxalic acid leaching process without the use of external oxidants for the semi‐selective recovery of lithium from industrial LFP black mass. Box‐Behnken response surface methodology (RSM) is employed to optimize key process parameters, including temperature, acid concentration, and solid‐to‐liquid ratio. In parallel, a comprehensive kinetic analysis is performed using pseudo‐first‐order, shrinking‐core, and Avrami models to identify the governing mechanism and rate‐controlling steps.

The novelty of this work lies in the integration of statistical optimization with comparative kinetic modeling using realistic industrial black mass under mild operating conditions, demonstrating that efficient lithium recovery can be achieved at low temperature. By linking process parameters to reaction mechanisms and selectivity behavior, this study provides fundamental insights and practical design criteria to support the development of resource‐efficient, scalable, and environmentally sustainable recycling technologies for LFP batteries

## 2 Materials and Methods

### 2.1 Materials

Envirostream Australia, a battery recycling company, provided the black mass from EoL LFP batteries used in this work. The black mass was characterized by ALS Metallurgy (Perth, WA) using metaborate fusion and XRF (Al, Fe, and P), and Ash/sodium peroxide fusion and inductively coupled plasma optical emission spectrometry (ICP‐OES, iCap PRO, Thermo Scientific, USA) for Li. The black mass composition is presented in Table 1. Carbon constitutes the largest fraction (50 wt.%), originating from graphite anode material and conductive carbon additives. The remaining 13.9 wt.% is attributed primarily to oxygen bound in the LiFePO_4_ phosphate structure, with minor contributions from binder residues and trace components not quantified by the analytical methods employed.

**TABLE 1:** Composition of commercial LFP black mass characterized by ALS and carbon (graphite).

Elements	Li	Fe	P	Al	Cu	C
Content (wt.%)	2.58	17.70	10.30	3.45	2.07	50

Chemical reagents used for the leaching experiments included oxalic acid (C_2_H_2_O_4_, ≥ 98%, analytical reagent grade, Sigma–Aldrich), hydrochloric acid (HCl, 37%, AR, Sigma–Aldrich), nitric acid (HNO_3_, 60–70 wt.%, AR, Sigma–Aldrich), and 10 000 ppm standard solutions of Li, Fe, P, Al and Cu (Chromatographically pure, ROWE SCIENTIFIC PTY LTD) for ICP‐OES analysis. All experiments were conducted using deionized water purified with a Milli‐Q water purification system (Millipore, USA).

### 2.2 Leaching Method

All leaching experiments were conducted in a 250 mL three‐necked round‐bottom flask. The central neck, connected to a reflux condenser, prevented the loss of volatile components and maintained a stable reaction environment. The side neck enabled periodic sampling during leaching without interrupting the process. The flask was immersed in a temperature‐controlled oil bath heated using a digital hot plate to ensure uniform thermal distribution. A magnetic stirrer provided homogeneous mixing of the slurry at a constant speed of 600 rpm.

For each experiment, the reaction mixture was continuously stirred, and samples were withdrawn at predetermined time intervals (0, 5, 10, 15, 30, 60, 90, 120, 150, and 180 min) through the side neck using a syringe. Each sample was immediately filtered using a 0.22 µm syringe filter. The resulting leachate was diluted with 2% HNO_3_ to stabilize metal ions and ensure compatibility with analytical techniques. Metal concentrations in diluted samples were quantified using ICP‐OES. The leaching efficiency of each element was calculated using the following Equation (1).

(1) $$\mathrm{Leaching}\;\;\mathrm{efficiency}\left(\%\right)=\frac{C\times V}{m\times w}\times 100$$

where C is the concentration of the element in leachate (g L^−1^), V is the volume of leachate (L), m is the mass of material (g), and w is the weight percentage of the element in raw material (wt.%).

### 2.3 Experimental Design and Statistical Modelling

The influence of operating parameters on lithium extraction efficiency was investigated using Response Surface Methodology (RSM) based on a three‐factor Box‐Behnken Design (BBD). The BBD was selected for its rotatability and efficiency in estimating second‐order models while requiring fewer experimental runs than a full factorial design. Specifically, the design required 15 runs compared with 27 for a three‐level full factorial, and it avoids the extreme factor combinations at the cube corners that can be experimentally impractical. A second‐order polynomial was considered appropriate because the response exhibits pronounced curvature. Lithium extraction peaks at intermediate oxalic acid concentration and declines at both higher and lower values, which a first‐order model cannot capture, with no higher‐order behavior evident within the studied ranges. ANOVA (Section 3.2) confirmed the adequacy of the second‐order model.

The independent variables examined were temperature (X_1_: 35°C, 50°C, 70°C), oxalic acid concentration (X_2_: 0.30, 0.65, 1.00 M), and solid‐to‐liquid ratio (X_3_: 20, 60, 100 g L^−1^). These three factors were selected because they control the principal physicochemical drivers of leaching: temperature determines the reaction driving force, acid concentration determines the availability of the leaching and precipitating reagent, and the solid‐to‐liquid ratio determines pulp density and the associated mass‐transfer conditions. They are also the parameters most readily manipulated at process scale and are the variables varied in previous studies of organic‐acid leaching of LFP black mass [44, 46, 47]. Stirring speed (600 rpm) and particle size (as‐received industrial black mass) were held constant. Factor levels were defined based on preliminary leaching trials and literature precedent [48].

The BBD consisted of 15 experimental runs, including 12 factorial edge points and 3 replicates at the centre point to estimate pure experimental error. The complete experimental matrix with coded and actual values is presented in Table 2.

**TABLE 2:** Box‐Behnken experimental design matrix with coded and actual factor levels.

Run	Type	X_1_	X_2_	X_3_	Temp (°C)	Acid (M)	S/L (g L^−1^)
01	Edge	−1	−1	0	35	0.30	60
02	Edge	−1	0	−1	35	0.65	20
03	Edge	−1	+1	0	35	1.00	60
04	Edge	−1	0	+1	35	0.65	100
05	Edge	0	−1	−1	50	0.30	20
06	Edge	0	+1	−1	50	1.00	20
07	Edge	0	−1	+1	50	0.30	100
08	Edge	0	+1	+1	50	1.00	100
09^a^	Centre	0	0	0	50	0.65	60
10^a^	Centre	0	0	0	50	0.65	60
11^a^	Centre	0	0	0	50	0.65	60
12	Edge	+1.33	−1	0	70	0.30	60
13	Edge	+1.33	+1	0	70	1.00	60
14	Edge	+1.33	0	−1	70	0.65	20
15	Edge	+1.33	0	+1	70	0.65	100

^a^ Centre point replicate was included to estimate experimental error and assess model precision. Independent variables were transformed into dimensionless coded values according to Equation (2).

(2) $$x_{i}=\frac{\left(X_{i}-X_{0}\right)}{\Delta X_{i}}$$

where x_i_ is the coded value, X_i_ is the actual value, X_0_ is the center point value, and ∆X_i_ is the step change value.

Acid concentration and solid‐to‐liquid ratio were varied symmetrically about their center points (0.65 ± 0.35 m and 60 ± 40 g L^−1^). The temperature levels (35°C, 50°C, and 70°C) were chosen on practical grounds and are not equally spaced about the center point; temperature was therefore scaled as X_1_ = (T−50)/15, placing 70°C at a coded value of +1.33.

The experimental data were fitted to a second‐order polynomial regression model as shown in Equation (3).

(3) $$Y=\beta_{0}+\sum \beta_{i}x_{i}+\sum \beta_{ii}x_{i}^{2}+\sum \beta_{i}x_{i}x_{j}+\varepsilon$$

where Y is the predicted response (Li extraction, %), β_0_ is the intercept coefficient, β_i_ are linear coefficients, β_ii_ are quadratic coefficients, β_ij_ are interaction coefficients, x_i_ and x_j_ are coded independent variables, and ε is the residual error.

Statistical significance of model terms was evaluated at the 95% confidence level (α = 0.05). Model adequacy was assessed using analysis of variance (ANOVA), including F‐statistics and p‐values. The goodness of fit was evaluated using the coefficient of determination (R^2^) and the adjusted coefficient of determination (R^2^_adj). Numerical optimization was performed using a desirability function approach to determine the operating conditions maximizing lithium extraction. Response surface and contour plots were generated to visualize effects.

All data analysis, regression modeling, and statistical evaluation were performed in Python (version 3.12).

### 2.4 Analytical Methods

Metal concentrations (Li, Fe, Al, Cu, and P) in filtered leachates were quantified by ICP‐OES. Calibration standards were prepared in 2 vol% HNO_3_ to match the sample matrix. Calibration curves exhibited R^2^ values based on triplicate measurements.

Phase composition of the solid samples (black mass and leaching precipitates) was analyzed by X‐ray diffraction (XRD) using a PANalytical Empyrean diffractometer operated at 40 kV and 40 mA with Co Kα radiation (λ = 0.1789 nm). Diffraction patterns were collected over a 2θ range of 5°–118° with an effective counting time of 58.4 s to ensure high‐resolution phase identification.

Phase identification was carried out using HighScore Plus software (Malvern Panalytical) in conjunction with the ICDD Powder Diffraction File (PDF) database. The LiFePO_4_ reference pattern (PDF 98‐018‐9057) was used for phase comparison. As the reference pattern is reported in Cu Kα coordinates, the peak positions were converted to Co Kα equivalents using Bragg's law prior to comparison.

Morphology and elemental distribution of solids were examined using a JEOL Neoscope JCM‐7000 benchtop scanning electron microscope (SEM) equipped with energy‐dispersive X‐ray spectroscopy (EDS).

### 2.5 Kinetic Modelling and Mechanism Analysis

To investigate the rate‐controlling mechanism of lithium leaching, time‐dependent extraction data were fitted to established kinetic models commonly applied in hydrometallurgical systems.

#### 2.5.1 Pseudo‐First‐Order Model

The Pseudo‐First‐Order (PFO) model assumes reaction‐controlled dissolution under excess reagent conditions as shown in Equation (4).

(4) $$E\left(t\right)=E_{max}\left(1-\exp\left(-kt\right)\right)$$

#### 2.5.2 Shrinking Core Model (Diffusion Control)

The leaching kinetics can be described by using the shrinking core model. For spherical particles with product layer diffusion control, the shrinking core model is expressed as shown in Equation (5) [49].

(5) $$1-3{\left(1-X\right)}^{\frac{2}{3}}+2\left(1-X\right)=k_{d}.dt$$

where X is the fractional conversion and k_d_ is the diffusion rate constant.

#### 2.5.3 Avrami (JMAK) Model

The Avrami equation described in Equation (6) is commonly used to describe nucleation and growth kinetics in heterogeneous systems [50].

(6) $$E\left(t\right)=E_{max}\left(1-\exp\left(-k_{a}t^{n}\right)\right)$$

where k_a_ is the rate constant, and n is the Avrami exponent. The magnitude of n is the Avrami exponent reflecting the nucleation and growth mechanism.

In the leaching of EoL LFP‐based batteries, the Avrami model was employed as an alternative kinetic description to the shrinking‑core model, accounting for the heterogeneous nature of black‐mass particles. The model treats metal dissolution as a time‑dependent transformation initiated at active surface sites and progressing through the growth of the reacted region, rather than assuming a sharp, uniformly advancing reaction interface.

In Equation (6), the Avrami exponent ‘n’ reflects the dominant leaching mechanism, analogous to the controlling step identified in the shrinking‑core model (e.g., surface chemical reaction or diffusion through a product layer). The apparent rate constant k_a_ represents the overall leaching rate under the given experimental conditions, accounting for intrinsic reaction kinetics, solution chemistry, and temperature.

#### 2.5.4 Activation Energy

Activation energy (E_a_) was determined using the Arrhenius equation, as shown in Equation (7) [49].

(7) $$\ln\left(k\right)=\ln\left(A\right)-E_{a}/RT$$

where K is the reaction rate constant, A is the pre‐exponential factor (the unit is the same as k), Ea is the apparent activation energy (J mol^−1^), T is the absolute temperature (K), and R is the universal gas constant (8.314 Jmol K^−1^). Temperature‐averaged rate constant (35°C, 50°C, 70°C) was plotted as ln(K) vs. 1/T, with activation energy calculated from the linear regression slope (−E_a_/R). According to established hydrometallurgical criteria, activation energies Below ~40 KJ mol^−1^ suggest chemical reaction‐controlled processes [51].

#### 2.5.5 Results and Discussion

### 2.6 Structure and Composition of Black Mass

XRD confirmed that the black mass consisted predominantly of crystalline LiFePO_4_ (Pnma, PDF 98‐018‐9057) and graphite (Figure S1). After oxalic acid leaching, the characteristic LFP reflections disappeared while graphite remained, confirming selective dissolution of the LiFePO_4_ phase. No crystalline iron oxalate was detected in the leach residues; however, ICP‐OES showed that only 14.1 ± 4.9% (Figure 2A) of the iron was in solution, indicating that most of the iron remained in the solid phase. The precipitated iron oxalate is therefore likely amorphous or poorly crystalline. It has been shown previously by XRD that lithium dissolves into solution, while iron precipitates as amorphous iron oxalate [52].

SEM‐EDS analysis (Figures S2 and S3) confirmed the morphology and elemental distribution of the black mass, with Fe and P as the principal components, consistent with the expected LiFePO_4_ stoichiometry (Table 1). Li is not detectable by EDS due to its low atomic number, but it was measured by ICP‐OES. Fragmented Al and Cu current collector particles were also identified, which may influence downstream hydrometallurgical selectivity and reagent consumption.

The run order and experimental conditions tested in the Box‐Behnken design, along with the corresponding metal extraction efficiencies at 180 min, are presented in Table 3. The effect of leaching time on the leaching efficiency was investigated across all experiments. Leaching of Li reached a plateau within 60–90 min for most experimental conditions, with extraction remaining stable up to 180 min. Runs at low acid concentration (0.30 M) or high S/L ratio (100 g L^−1^) required up to 120–150 min to stabilize, consistent with the slower kinetics expected under acid‐limited conditions. Hence, 180 min was selected as the endpoint for RSM and kinetic analysis to ensure all conditions had reached equilibrium. Leaching efficiency graphs at all time points are provided in Figure S4.

**TABLE 3:** Leaching efficiencies (%) of target elements obtained from LFP black mass based on Box‐Behnken design experiments at 180 min.

Run	T (°C)	Acid (M)	S/L (g L^−1^)	Type	Li (%)	Al (%)	Fe (%)	P (%)	Cu (%)
01	35	0.30	60	Edge	94.1	96.8	15.6	100	7.8
02	35	0.65	20	Edge	91.5	91.7	16.7	100	6.0
03	35	1.00	60	Edge	100	96.8	11.5	100	9.0
04	35	0.65	100	Edge	78.1	77.4	9.9	100	1.6
05	50	0.30	20	Edge	89.8	94.7	16.3	100	6.5
06	50	1.00	20	Edge	93.0	96.8	20.6	100	9.9
07	50	0.30	100	Edge	38.5	47.6	9.8	46.1	4.7
08	50	1.00	100	Edge	54.1	55.5	5.9	60.0	2.9
09	50	0.65	60	Center	98.7	96.8	7.5	100	4.3
10	50	0.65	60	Center	98.3	96.8	13.0	100	6.0
11	50	0.65	60	Center	99.5	96.8	20.8	100	10.6
12	70	0.30	60	Edge	88.6	85.2	16.1	100	3.2
13	70	1.00	60	Edge	94.8	88.1	18.7	100	5.7
14	70	0.65	20	Edge	81.7	68.5	20.0	100	10.5
15	70	0.65	100	Edge	73.3	63.9	9.2	100	6.8

Li extraction ranged from 38.5% to 100%, demonstrating substantial sensitivity to operating conditions within the investigated parameter space. Center‐point replicates (Runs 09, 10 and 11) yielded 99.5 ± 0.6% lithium extraction (RSD = 0.6%), confirming high experimental precision and reproducibility.

The experimental data were initially fitted to a full second‐order polynomial model containing all nine terms (three linear, three interactions, and three quadratic). The full model was marginally non‐significant (*p* = 0.081), with an adjusted R^2^ of 0.637, indicating substantial overfitting given that nine parameters were estimated from 15 observations. Backward elimination was therefore applied, sequentially removing the least significant term at each step (threshold *p* > 0.10) while maintaining model hierarchy (i.e., retaining parent main effects whenever their corresponding quadratic or interaction terms remained in the model). All temperature‐related terms (T, T^2^, T x Acid, T x S/L) and all interaction terms were non‐significant and eliminated, yielding a reduced four‐term model including S/L and acid conc. related terms (Table 4) that were highly significant (*p* = 0.0008), with an improved adjusted R^2^ = 0.756. The regression coefficients for the full and reduced models can be found in Tables S1 and S2.

**TABLE 4:** ANOVA results for the reduced RSM of Li leaching.

Source	Sum of squares(SS)	Degrees of freedom(df)	Mean square (MS)	F‐value	p‐value	Significance level
Model	3663.65	4	915.91	11.82	0.0008	^****^
x_2_ (Acid)	118.99	1	118.99	1.54	0.244	N.S.
X_3_ (S/L)	1568.99	1	1568.99	20.25	0.001	^***^
x_2_^2^	278.95	1	278.95	3.60	0.087	^*^
X_3_^2^	1787.52	1	1787.52	23.07	< 0.001	^****^
Residual	774.79	10	77.48			
Lack of fit	774.00	8	96.75	243.96	0.004	^****^
Pure error	0.79	2	0.40			
Total	4438.44	14				

^*^ *p* < 0.10, ^**^ *p* < 0.05, ^***^ *p* < 0.01, ^****^ *p* < 0.001, N.S. = non‐significant.

The four‐term model is given by Equation (8).

(8) $$Y=101.25+3.86x_{2}-14x_{3}-8.67x_{2}^{2}-21.94$$

The statistically significant lack‐of‐fit (p = 0.004) is attributed to the exceptionally high precision of center‐point replicates (RSD = 0.6%), which produces a very small pure error estimate (SS_PE = 0.79, 2 df). Under these conditions, non‐significant LOF would require R^2^ > 0.998, exceeding the capability of any second‐order polynomial model.

Although the acid concentration linear term was not individually significant (*p* = 0.244), its quadratic counterpart (x_2_^2^, *p* = 0.087) was retained at the 0.10 threshold used during backward elimination, and the linear term was retained, preserving the model hierarchy. The inclusion of both acid terms improved the adjusted R^2^ from 0.692 to 0.756, and the curvature is physically meaningful, with extraction peaks near 0.71 M, declining at both lower concentrations (insufficient reagent) and higher concentrations, where excess oxalate promotes early iron oxalate precipitation that can seal the surfaces of unreacted black mass. This behavior is consistent with [53], who observed lithium extraction plateau at 0.3 M then decrease to 86% at 1.5 M oxalic acid, and with [54], who reported that supersaturated oxalic acid (2.5 M) reduced lithium recovery from > 90% to 72% due to extensive precipitation of metal‐oxalate complexes that hindered further dissolution.

The observed vs. predicted plot (Figure S5) shows a coefficient of determination (R^2^) of 0.825 for the reduced model. This is lower than the R^2^ = 0.99 reported by [55] for oxalic acid leaching of NMC black mass using a comparable Box–Behnken design with real industrial black mass. Two factors account for this difference. First, the present model was deliberately kept parsimonious: backward elimination reduced the full nine‐term polynomial (R^2^ = 0.882) to a four‐term model to avoid overfitting, whereas [55] retained 10 regression terms for 18 observations. Second, the black mass‐oxalic acid system involves tightly coupled dissolution and precipitation at the same particle surface, as dissolved Fe^2^^+^ immediately reprecipitates as FeC_2_O_4_·2H_2_O, forming a passivation layer whose extent depends on local particle morphology and solution saturation conditions that a quadratic polynomial cannot fully capture. Accordingly, the largest residuals (Runs 04, 07, 08, 15) all occur at high S/L ratios, where particle packing intensifies mass‐transfer limitations. Despite the moderate R^2^, the model is highly significant (*p* = 0.0008) and adequately predicts the main process trends for RSM optimization.

### 2.7 Response Surface Analysis and Optimization

Three‐dimensional response surface plots (Figure 1) illustrate the relative influence of the investigated parameters.

**FIGURE 1:**
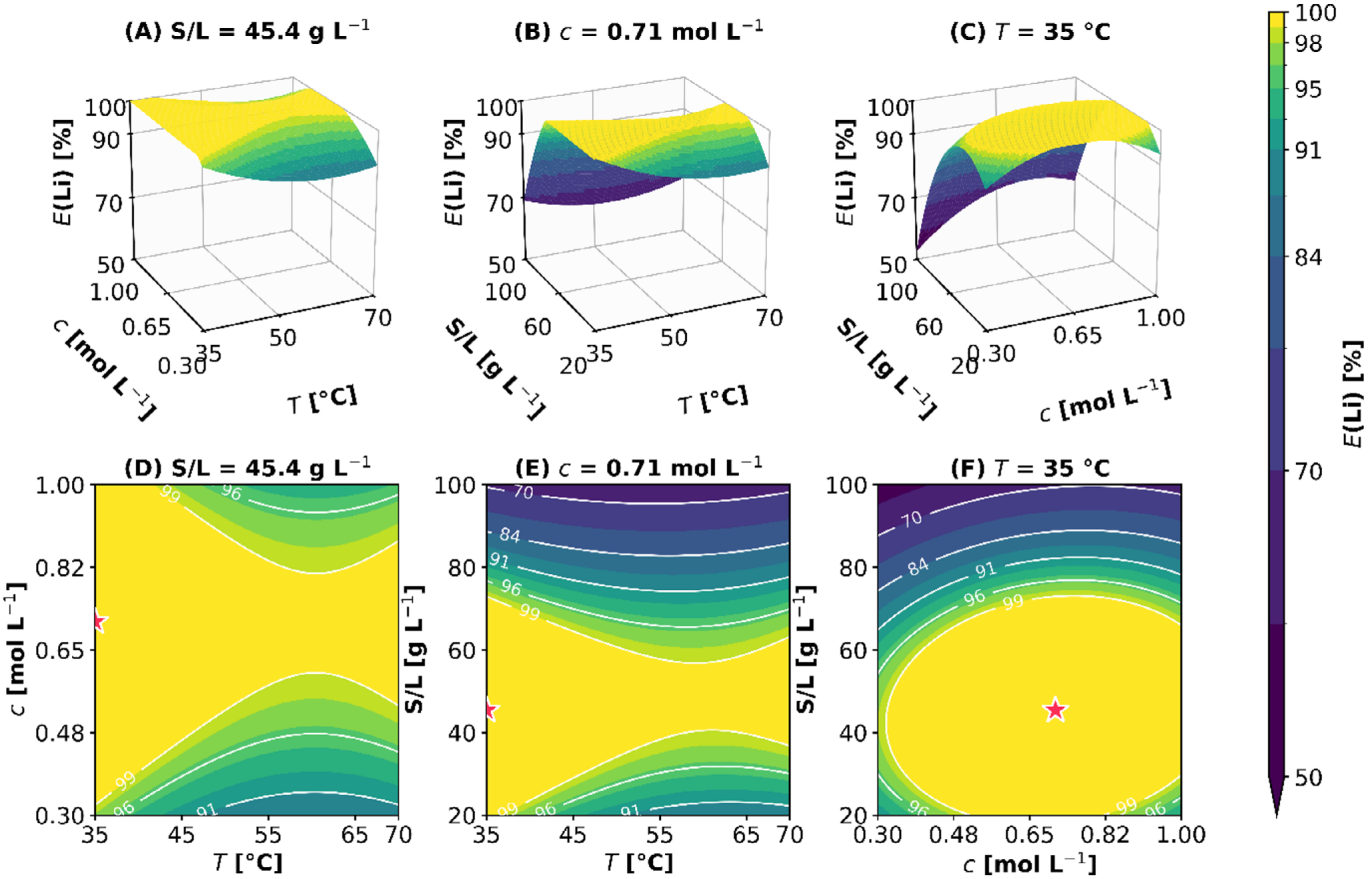
Response surface analysis for lithium extraction efficiency at 180 min. Top row (A–C): three‐dimensional surface plots; bottom row (D–F): corresponding contour plots with optimal conditions marked (★). (A, D) Temperature vs. acid concentration at S/L = 45.4 g L^−1^; (B, E) Temperature vs. S/L ratio at acid = 0.71 M; (C, F) Acid concentration vs. S/L ratio at temperature = 35°C.

Figure 1A (temperature vs. acid concentration at S/L = 45.4 g/L) exhibits a relatively flat surface, indicating modest sensitivity to these parameters. In contrast, Figure 1B (temperature vs. S/L ratio at 0.71 M acid) shows a pronounced decline in extraction with increasing S/L ratio, confirming its dominant effect. Figure 1C (acid concentration vs. S/L ratio at 35°C) shows that increasing acid concentration partially mitigates the negative effect of higher solid content but does not fully compensate for elevated S/L ratios, as also indicated by the high p‐values for acid concentration in the model fit, as discussed in Section 3.2.

Contour plots define the optimal operating region. Numerical optimization predicted maximum lithium extraction at 35°C, 0.71 M oxalic acid, and an S/L ratio of 45.4 g L^−1^, corresponding to full predicted extraction (Figure 1D–F). Experimental validation under these conditions achieved 96% lithium extraction with 6% iron co‐extraction, confirming the model's predictive capability and the effectiveness of the selectivity mechanism under optimized operating conditions. Contour plots fitted independently at 30, 60, and 120 min (Figure S6) show that the S/L‐dominated pattern and optimal region are consistent across leaching times, with the model fitting best at 30 min (R^2^ = 0.95). Specifically, the optimal temperature lies at the lower boundary of the investigated range, suggesting that elevated temperatures are not required to achieve high extraction efficiency. From a process perspective, operation near ambient temperature offers potential energy advantages compared to thermally driven mineral acid systems.

### 2.8 Leaching Results and Selectivity Analysis

Mean extraction efficiencies were Li (84.9 ± 17.2%), Al (83.6 ± 16.3%), Fe (14.1 ± 4.8%), P (93.7 ± 16.2%), and Cu (6.4 ± 2.7%), as shown in Figure 2A. Selectivity ratios were calculated from the mean values to be Li/Fe = 6.62, Li/Al = 1.01, Li/p = 0.90, Li/Cu = 16.73 (Figure 2B). Excellent Li/Fe selectivity (6.63) confirms effective Fe rejection through iron oxalate precipitation, the intended mechanism of oxalic acid leaching. The low Fe extraction (14.1%) indicates that > 85% of iron is removed from solution as a precipitate, thereby simplifying downstream processing. A high Li/Cu selectivity (16) shows that most of the Cu also ends up in the precipitate. Figure 2C visualizes the leaching efficiency for each run.

**FIGURE 2:**
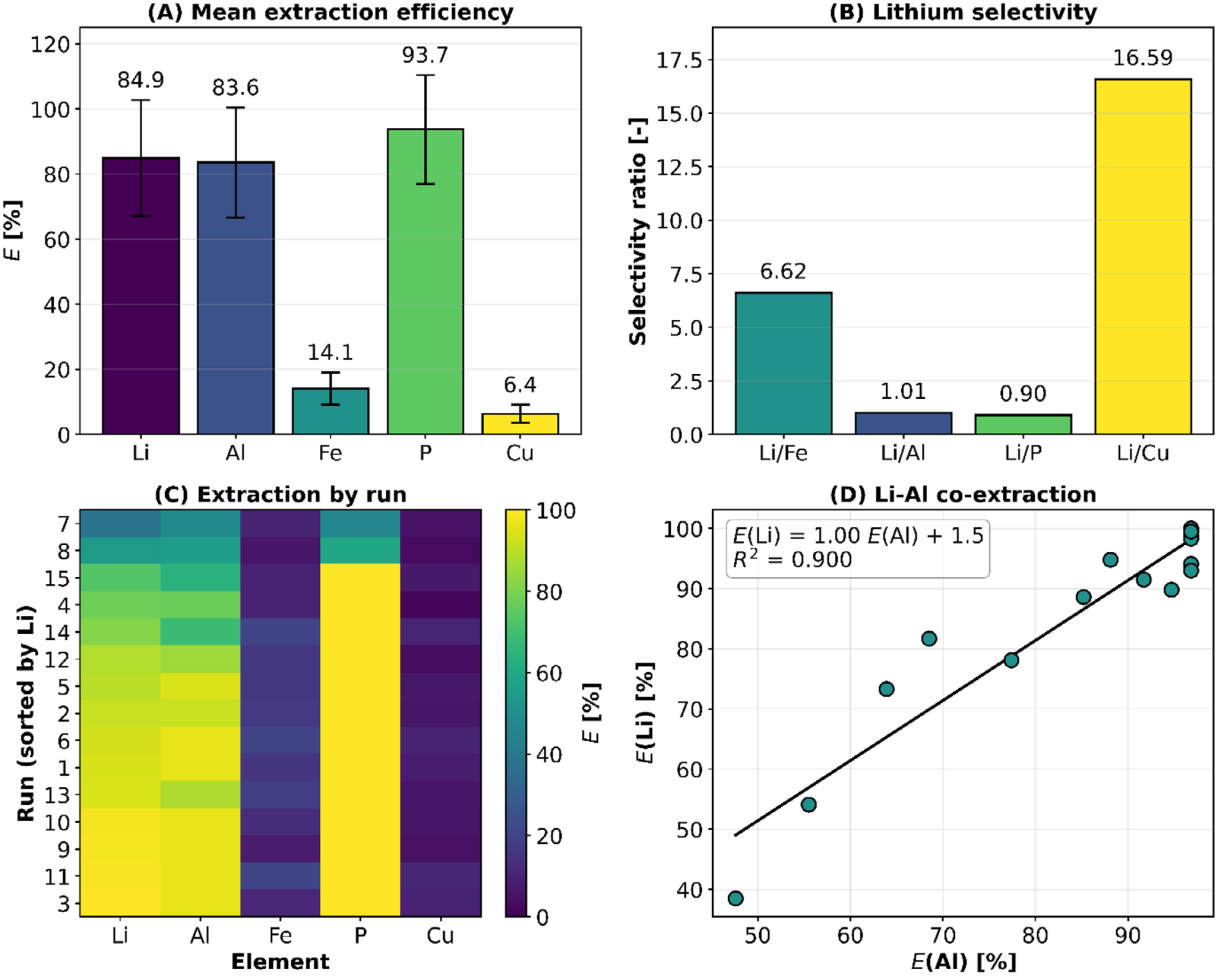
Metal extraction performance and selectivity analysis at 180 min. (A) Mean extraction efficiencies of Li, Fe, P, Al, and Cu (error bars represent standard deviation). (B) Lithium selectivity ratios relative to co‐existing metals. (C) Heatmap of metal extraction efficiencies across experimental runs (sorted by Li extraction). (D) Correlation between Li and Al extraction.

Poor Li/Al selectivity (1.01) indicates that aluminum co‐extracts in nearly the same proportion as lithium. The strong linear correlation between Li and Al extraction (R^2^ = 0.900; Figure 2D) demonstrates that this systematic co‐extraction behavior poses a critical challenge requiring downstream purification. Similar leaching behavior has been reported in the literature, including oxalic acid leaching of real industrial black mass from NMC cathode material [55] and for mixed LFP/NMC [54]. Aluminum separation will require additional unit operations, such as solvent extraction with organophosphorus extractants (e.g., D2EHPA), selective precipitation via pH adjustment, or separation using, e.g., membrane filtration or ion exchange. This adds complexity and cost to the process that must be considered in economic analysis.

Phosphorus shows the highest extraction efficiency (93.7%) with a Li/P selectivity ratio of 0.87, indicating preferential dissolution of phosphorus relative to lithium. This near‐complete phosphorus extraction confirms thorough breakdown of the LiFePO_4_ olivine structure during leaching. While complete P dissolution is favorable for maximizing lithium recovery (no Li trapped in residual phosphate phases), the high phosphorus content in the leachate (P/Li molar ratio ∼3.2:1 based on black mass composition) necessitates phosphorus removal for production of battery‐grade lithium carbonate. Phosphorus can also be used to produce Li_3_PO_4_ from the leachate and has potential for use in producing new battery materials [56].

### 2.9 Leaching Chemistry Monitoring

Real‐time pH and conductivity monitoring across all experiments provided a mechanistic understanding of process limitations (Figure 3).

**FIGURE 3:**
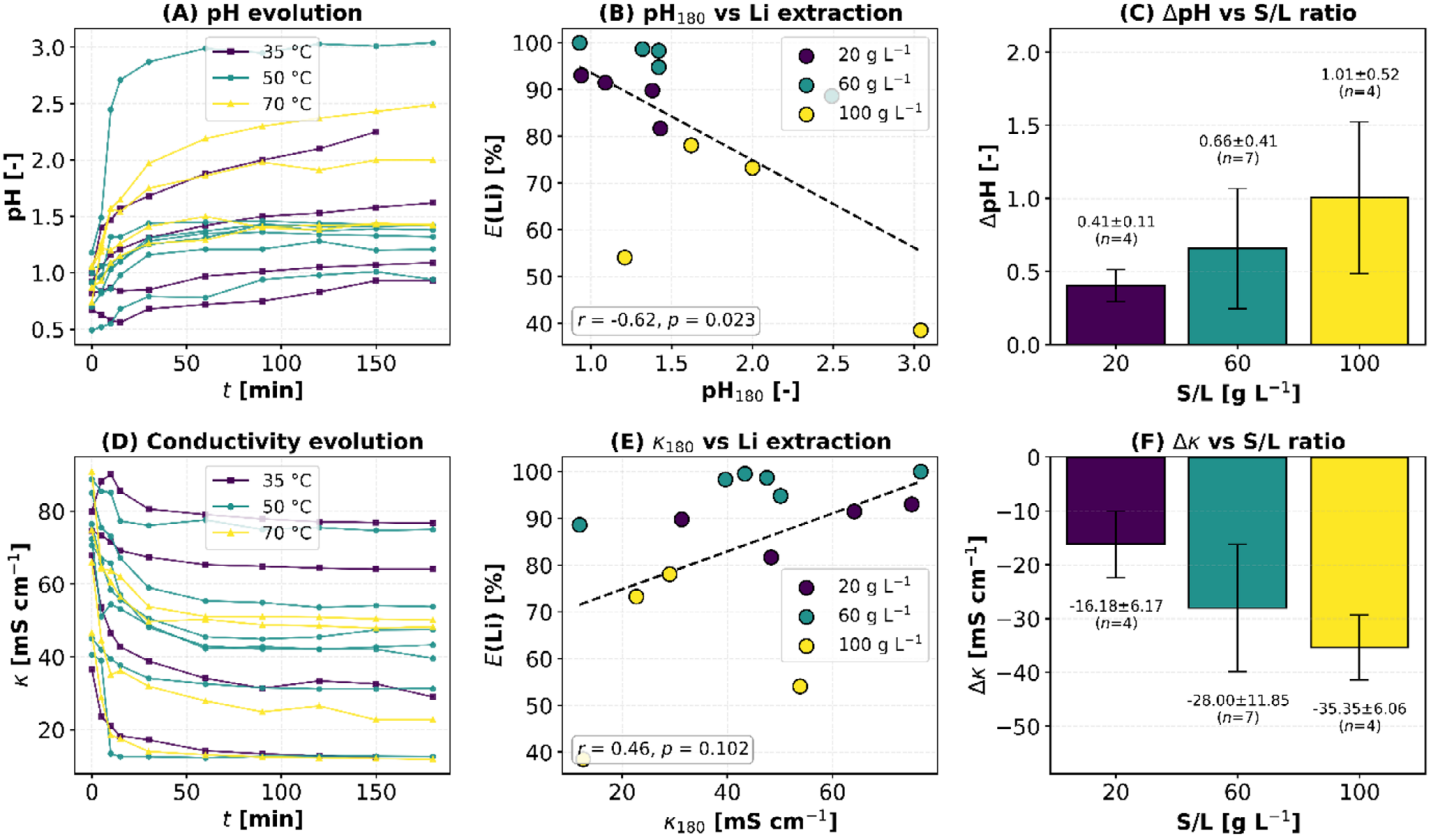
pH (A–C) and conductivity (D–F) monitoring during leaching. Left panels (A, D): time‐dependent profiles for all runs, coloured by temperature. Centre panels (B, E): correlation with lithium extraction efficiency at 180 min, coloured by S/L ratio with acid concentration labels. Right panels (C, F): mean change (±SD) as a function of S/L ratio.

Sequential evolution (Figure 3A) shows a rapid initial increase in pH over the first 30 min, corresponding to the fastest extraction, followed by a gradual, sustained rise. pH increase indicates progressive acid consumption through proton transfer in dissolution reactions and buffering by released phosphate species.

At 35°C (purple), runs show modest pH increases (ΔpH = 0.26–0.70), while at 50°C (green), a single run (Run 7) rises sharply to pH 3.04 (ΔpH = 1.86). The 70°C (yellow) runs show intermediate behavior (ΔpH = 0.56–1.43). No systematic temperature trend is evident, consistent with S/L rather than temperature governing acid depletion. Higher solid loading yields larger pH increases (Figure 3C): Mean ΔpH rose from 0.41 at 20 to 0.66 at 60 g L^−1^ and 1.01 at 100 g L^−1^, indicating greater acid depletion.

Critically, the final pH at 180 min (pH_180_) showed a significant negative correlation with lithium extraction (r = −0.62, *p* = 0.023, Figure 3B), indicating that acid depletion accounts for approximately 39% of the observed variation in extraction efficiency. The remaining variance reflects the direct influence of other BBD factors (acid concentration, S/L ratio) that are not fully captured by the pH metric alone. Nevertheless, the correlation is consistent with excessive acid depletion limiting extraction. A higher pH at the end of leaching (180 min) indicates substantial proton consumption, thereby reducing the driving force for continued dissolution of EoL LFP‐based battery black mass. This observation is consistent with [57], who found that final solution pH had a clear impact on iron leaching yield in mild‐acid LFP systems, and with Henderson, Beh, Oraby and Eksteen [48], who demonstrated that leaching selectivity shifts fundamentally across the pH 0.5–4 range in oxalic acid systems. While the negative effect of acid depletion is widely recognized qualitatively [52], this study provides the quantitative statistical correlation between final pH and lithium extraction across a systematic BBD experimental design. All six runs with ΔpH < 0.5 achieved 89%–100% lithium extraction, whereas larger pH increases gave more variable and generally lower extraction (38%–95%). The lowest extraction (38.5%) corresponded with the largest pH increase (run 7, ΔpH = 1.86, pH_180_ = 3.04) at 100 g L^−1^. A small pH rise is therefore necessary but not sufficient for high extraction; for example, Run 8 showed ΔpH = 0.51 yet only 54.1% extraction, reflecting the independent effect of high solid loading, so ΔpH is best used as an indicator that acid depletion is not limiting, rather than as a stand‐alone guarantee of yield.

Conductivity decreased consistently, with a mean reduction of 26.7 ± 12.1 mS cm^−1^ (40.7% relative), ranging from −43.3 to −3.3 mS cm^−1^. This systematic decrease confirms the intended selectivity mechanism; extensive iron oxalate precipitation removes both Fe^2^^+^ cations and C_2_O_4_^2−^ anions from solution [52, 55], reducing ionic strength and conductivity as shown in Equation (9).

(9) $$Fe^{2+}+C_{2}O_{4}^{2-}+2H_{2}O\to \mathrm{Fe}C_{2}O_{4}\cdot 2H_{2}O↓$$

Temporal evolution (Figure 3D) shows a rapid initial drop in 15–30 min (fast iron precipitation), followed by a gradual continued decrease. Unlike pH, conductivity showed no significant correlation with Li extraction (*r* = 0.46, *p* = 0.102; Figure 3E). This lack of correlation is mechanistically expected; conductivity primarily reflects iron precipitation rather than lithium dissolution. Iron removal occurs rapidly and extensively across all conditions (> 85% Fe rejection, Table 3), regardless of Li extraction efficiency. The weak positive trend may reflect that conditions that favor iron precipitation also favor lithium dissolution. Figure 3F shows the effect of S/L. Bars colored by magnitude reveal that runs with large decreases (red and orange bars, > 30 mS cm^−1^) had high initial conductivities and high S/L ratios, while small decreases (blue bars, < 20 mS cm^−1^) occurred at low S/L with lower initial iron concentrations.

Nevertheless, conductivity monitoring provides valuable process control information. The consistent 40% decrease confirms that the selectivity mechanism functions as intended; iron is removed while lithium remains dissolved. This real‐time feedback could be implemented in industrial processes to verify proper iron precipitation without requiring offline analysis.

To identify the rate‐controlling mechanism, time‐series data from all 15 runs were fitted to three mechanistic models: pseudo‐first‐order (PFO), shrinking core with diffusion control (SCD), and Avrami (JMAK).

Figure 4A–C shows representative fits at three contrasting conditions, evaluated by RMSE and AIC. At a high S/L ratio (35°C, 0.65 M, 100 g L^−1^; Figure 4A), extraction stagnates near 78% after 60 min, and PFO systematically overpredicts the approach to equilibrium (RMSE = 7.11). The Avrami model captures the rapid onset followed by near‐complete stagnation (RMSE = 3.27), with an exponent of *n* = 0.14 indicating severe diffusion limitation. At center‐point conditions (50°C, 0.65 M, 60 g/L; Figure 4B), extraction reaches ∼98% by 180 min, and all models perform adequately, though the Avrami model remains superior (RMSE = 1.77 vs. 6.11 for PFO). The fitted exponent (*n* = 0.43) suggests mixed diffusion–reaction control under these moderate conditions. At low acid concentration (35°C, 0.30 M, 60 g L^−1^; Figure 4C), extraction reaches ∼94% by 90 min and plateaus. Again, the Avrami model provides the best description (RMSE = 2.96, *n* = 0.20), capturing the transition from rapid surface dissolution to diffusion‐limited behavior that the single‐parameter PFO and SCD models cannot reproduce.

**FIGURE 4:**
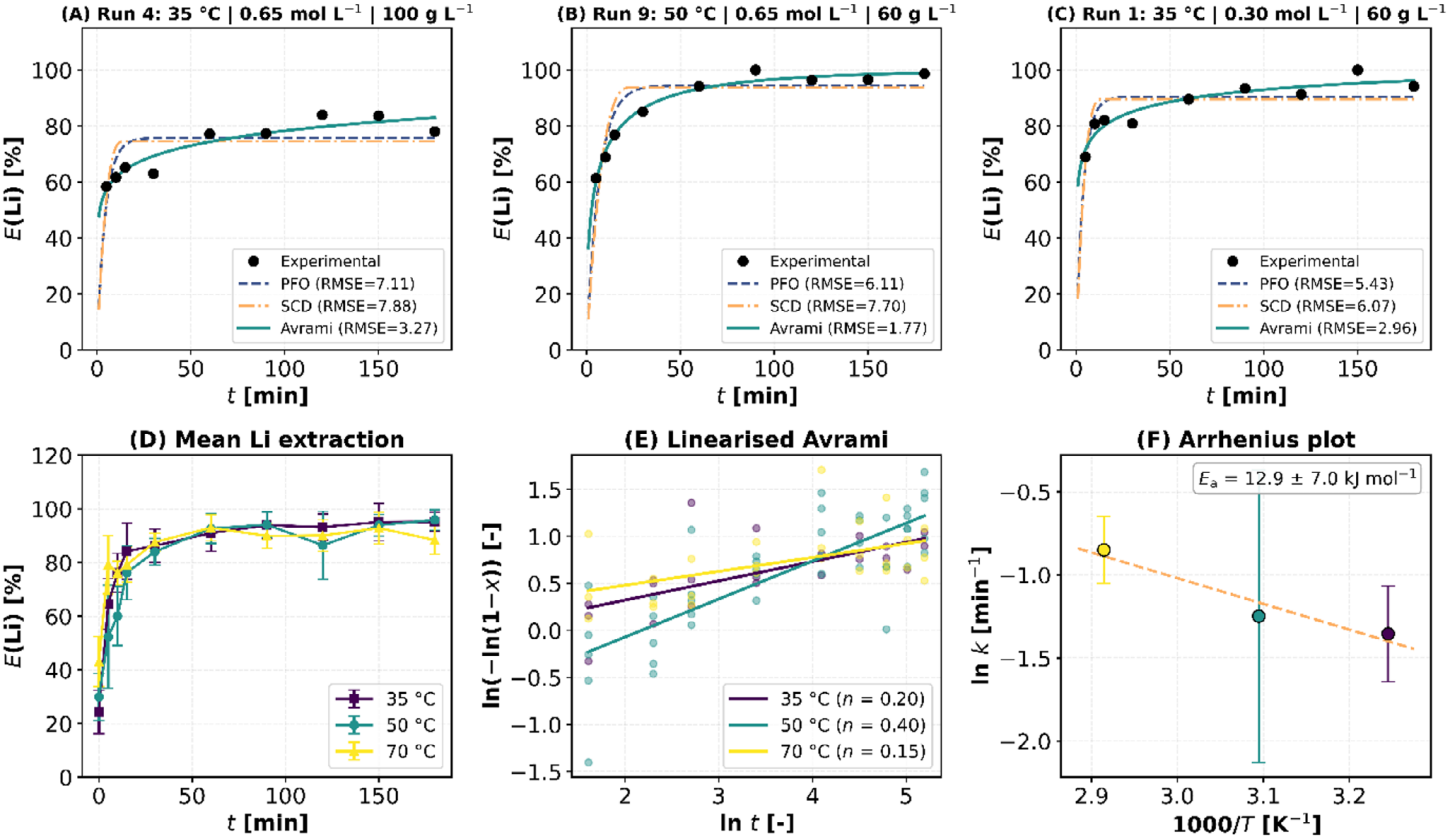
Kinetic analysis of lithium leaching. Top row (A–C): kinetic model comparison for three representative runs: (A) Run 4 (high S/L), (B) Run 9 (centre point), (C) Run 1 (low acid); solid line = Avrami (best fit by AIC), dashed = PFO, dash‐dot = SCD. Bottom row: Arrhenius analysis (S/L = 100 g/L excluded). (D) Mean Li extraction vs time at different temperatures. (E) Linearised Avrami plots; slopes correspond to exponent n. (F) Arrhenius plot yielding E_a = 12.9 ± 7.0 kJ mol^−1^. Error bars represent the standard deviation.

Across all 15 runs, the Avrami model emerged as the best‐performing model with a mean R^2^ = 0.763 ± 0.264 and 73% of runs achieving good fits (R^2^ > 0.7), compared to the SCD model (mean R^2^ = 0.616, 47% good fits) and PFO model (mean R^2^ = 0.592, 47% good fits) (Table S3). The Avrami exponent varied systematically with conditions (mean n = 0.57 ± 0.35, range 0.05–1.57), with lower values at high S/L ratios confirming increased diffusion resistance (Tables S4 and S5).

Arrhenius analysis was performed using temperature‐averaged Avrami rate constants to determine the apparent activation energy (Figure 4D–F). Runs conducted at S/L = 100 g L^−1^ were excluded from this analysis. At this high solid loading, acid is consumed faster than it can sustain the reaction, as evidenced by failures of the kinetic model and pH/conductivity monitoring under these conditions. The resulting reagent limitation suppresses rate constants independently of temperature and would bias the activation energy estimate. After exclusion, 12 runs remained across three temperature levels (*n* = 3, 5, and 3 at 35, 50, and 70°C).

Figure 4D shows that the mean extraction curves at different temperatures are nearly overlaid, reaching equivalent final extraction within 60 min regardless of temperature. The Avrami representation (Figure 4E) pools all filtered runs at each temperature, and the slopes correspond to the Avrami exponent n. The consistently low values (0.18–0.39) across all temperatures indicate diffusion‐dominated kinetics, and the near‐identical slopes and intercepts confirm that neither the reaction mechanism nor the rate changes appreciably from 35°C to 70°C.

The Arrhenius regression (Figure 4F) yielded mean rate constants that increased modestly with temperature (k_3__5_ = 0.258, k_5__0_ = 0.287, k₇_0_ = 0.428 min^−1^), corresponding to an apparent activation energy of 12.9 ± 7.0 kJ mol^−1^ (R^2^ = 0.928, p = 0.173). The regression did not reach statistical significance at α = 0.05, which is expected given only three temperature levels and the substantial within‐temperature variability inherent to the BBD design, particularly at 50°C, where five runs span different acid‐concentration and S/L ratio combinations. This variability reflects the dominant influence of these other process factors rather than measurement uncertainty, consistent with the RSM finding that S/L ratio controls extraction. Despite the limited statistical power, the E_a_ estimate of 12.9 kJ mol^−1^ falls below the 20 kJ mol^−1^ threshold conventionally associated with diffusion‐controlled processes [49].

This finding is consistent with the RSM 3D surface and contour plots, which show a flat temperature response across the entire design space (Section 3.2), the elimination of temperature as non‐significant during backward model reduction, and the Avrami exponent (mean *n* = 0.47), which independently indicates diffusion‐dominated kinetics. The weak temperature effect is not a limitation of the experimental design but a genuine characteristic of the system; temperature has minimal influence on oxalic acid leaching of EoL LFP‐based battery black mass because the rate‐limiting step is mass transport through the iron oxalate product layer rather than a thermally activated surface reaction.

Among reported LFP leaching, Hu, Yao, Meng, Li, Zhang, Zhang, Yang, Xu and Hu [58] found E_a_ = 18.69 kJ mol^−1^ for potassium pyrosulfate/H_2_O_2_ leaching of spent LFP using the Avrami model, classifying the process as mixed chemical‐diffusion control. Another study found an E_a_ = 31.96 kJ mol^−1^ for the H_2_SO_4_/oxalic acid leaching of NMC [59]. The present value (12.86 kJ mol^−1^) is consistent with these diffusion‐limited systems and substantially lower than that for chemically controlled processes, reflecting the unique dual role of oxalic acid as both leaching agent and precipitant, which creates a continuously growing iron oxalate product layer that governs mass transport.

## 3 Conclusions

This study demonstrates a low‐temperature oxalic acid process without external oxidants for the semi‐selective recovery of lithium from industrial EoL LFP black mass, integrating process optimization and kinetic modeling to provide both mechanistic insight and practical design guidance. Near‐complete extraction of lithium (96%) was achieved under mild conditions (35°C, 0.71 M oxalic acid, and an S/L ratio of 45.4 g L^−1^), confirming the feasibility of energy‐efficient operation, with extraction essentially complete within the range of 60–90 min. The solid‐to‐liquid ratio was identified as the dominant parameter influencing extraction efficiency, while temperature and acid concentration showed comparatively weaker effects within the investigated ranges. Kinetic analysis revealed a mixed diffusion‐reaction‐controlled mechanism governed by the precipitation of iron as amorphous iron oxalate, which rejects iron from solution and forms a product layer that restricts mass transfer, enabling favorable lithium‐iron separation (Li/Fe = 6.6). However, co‐extraction of aluminum and phosphorus (Li/Al = 1.0, Li/p = 0.9) indicates the need for downstream purification to achieve battery‐grade products. By addressing the lack of integrated optimization and kinetic understanding for industrial LFP black mass, this work demonstrates that efficient lithium recovery can be achieved under mild conditions and provides a scalable, resource‐effective, and environmentally sustainable framework for the development of industrial LFP recycling processes.

## Author Contributions

A.A.: conceptualization, methodology, investigation, validation, formal analysis, data curation, visualization, writing – original draft, writing, review and editing. M.R.A.: supervision, project administration, writing, review, and editing. A.R.: writing, review, and editing. S.P.: writing, review, and editing. Y.A.: writing, reviewing, and editing. Declaration of generative AI and AI‐assisted technologies in the manuscript preparation process.

## FUNDING

The research received no specific grant from any funding agency in the public, commercial, or not‐for‐profit sectors.

## Conflicts of Interest

The authors declare no conflicts of interest.

## Supporting information




**Supporting File 1**: gch270173‐sup‐0001‐SuppMat.png.

**Supporting File 2**: gch270173‐sup‐0002‐SuppMat.docx.

## Data Availability

The data that support the findings of this study are available from the corresponding author upon reasonable request.
