# Supplementary material for: Resource Recovery From Spent Lithium Iron Phosphate Batteries via Low‐Temperature Lithium Extraction
Source: Glob Chall. 2026 Nov 13;10(11):e70173. doi: 10.1002/gch2.70173 (PMC13768463; doi:10.1002/gch2.70173)
Supplement: Supplementary file 1 [file GCH2-10-e70173-s001.docx]

**Resource recovery from spent lithium iron phosphate batteries via low-temperature lithium extraction**

Asad Ali^a,b^, Yasir Arafat^a^, Sandra Pavón^c^, Amir Razmjou^a,d^, Muhammad Rizwan Azhar^a*^

^a^ School of Engineering, Edith Cowan University, 270 Joondalup Drive, Joondalup, WA 6027, Australia

^b^ Department of Chemical Engineering, University of Gujrat, Gujrat 50700, Pakistan

^c^ Fraunhofer Institute for Ceramic Technologies and Systems IKTS, Fraunhofer Technology Center for High-Performance Materials THM, 09599, Freiberg, Germany

^d^Mineral Resource Recovery Centre, Edith Cowan University, 270 Joondalup Drive, Joondalup, WA 6027, Australia

*Corresponding author [m.azhar@ecu.edu.au](mailto:m.azhar@ecu.edu.au)

*Table S1. Regression Coefficients for the Full Model*

| 1 | β (Coefficient) | SE | t | p | Sig |
| --- | --- | --- | --- | --- | --- |
| β₀ (Intercept) | 98.85 | 6.20 | 15.95 | <0.001 | **** |
| β₁ (Temperature) | -3.80 | 3.66 | -1.04 | 0.347 | N.S. |
| β₂ (Acid) | 3.86 | 3.81 | 1.01 | 0.358 | N.S. |
| β₃ (S/L ratio) | -14.18 | 3.81 | -3.72 | 0.014 | ** |
| β₁₂ | -0.03 | 4.58 | -0.01 | 0.995 | N.S. |
| β₁₃ | 2.09 | 4.58 | 0.46 | 0.667 | N.S. |
| β₂₃ | 3.09 | 5.37 | 0.58 | 0.589 | N.S. |
| β₁₁ | 3.27 | 4.21 | 0.78 | 0.472 | N.S. |
| β₂₂ | -8.37 | 5.59 | -1.50 | 0.194 | N.S. |
| β₃₃ | -21.64 | 5.59 | -3.87 | 0.012 | ** |

*p< 0.10, **p < 0.05, ***p < 0.01, ****p < 0.001, N.S. = Non-significant

*Table S2. Regression Coefficients for Reduced Model*

| Term | β (Coefficient) | SE | t-value | p-value | Sig. |
| --- | --- | --- | --- | --- | --- |
| β₀ (Intercept) | 101.25 | 4.23 | 23.95 | <0.001 | **** |
| β₂ (Acid) | 3.86 | 3.11 | 1.24 | 0.244 | N.S. |
| β₃ (S/L) | −14.00 | 3.11 | −4.50 | 0.001 | *** |
| β₂₂ (Acid²) | −8.67 | 4.57 | −1.90 | 0.087 | * |
| β₃₃ (S/L²) | −21.94 | 4.57 | −4.80 | <0.001 | **** |

*p< 0.10, **p < 0.05, ***p < 0.01, ****p < 0.001, N.S. = Non-significant

Table S3. Comparison of kinetic model fits using R^2^ and AIC criteria

| Model | Mean R² | Good fits (R² > 0.7) | Best by AIC |
| --- | --- | --- | --- |
| PFO | 0.592 ± 0.271 | 7/15 (47%) | 2/15 |
| SCD | 0.348 ± 0.367 | 3/15 (20%) | 1/15 |
| Avrami | 0.761 ± 0.267 | 11/15 (73%) | 12/15 |

*Table S4. Model fit for all runs*

| Experimental Conditions | | | | Pseudo-First-Order (PFO) | | | | | Shrinking Core Diffusion (SCD) | | | | | Avrami (JMAK) | | | | | | Best Model |
| --- | --- | --- | --- | --- | --- | --- | --- | --- | --- | --- | --- | --- | --- | --- | --- | --- | --- | --- | --- | --- |
| Run | **T (°C)** | **Acid (M)** | **S/L (g/L)** | **qe (%)** | **k (min⁻¹)** | **R²** | **RMSE** | **AIC** | **qe (%)** | **kd (min⁻¹)** | **R²** | **RMSE** | **AIC** | **qe (%)** | **k (min⁻¹)** | **n** | **R²** | **RMSE** | **AIC** |  |
| 77 | 35 | 0.30 | 60 | 90.1 | 0.2604 | 0.626 | 5.43 | 34.5 | 91.1 | 0.0532 | 0.629 | 5.41 | 34.4 | 108.1 | 0.3079 | 0.20 | 0.889 | 2.96 | 25.5 | Avrami |
| 78 | 35 | 0.65 | 20 | 86.1 | 0.1535 | 0.922 | 3.45 | 26.3 | 87.1 | 0.0305 | 0.957 | 2.55 | 20.8 | 88.1 | 0.1654 | 0.61 | 0.977 | 1.87 | 17.2 | Avrami |
| 79 | 35 | 1.00 | 60 | 99.1 | 0.2396 | 0.861 | 3.45 | 26.3 | 99.1 | 0.0511 | 0.909 | 2.79 | 22.5 | 100.1 | 0.3011 | 0.63 | 0.919 | 2.64 | 23.5 | SCD |
| 80 | 35 | 0.65 | 100 | 76.1 | 0.2286 | 0.427 | 7.11 | 39.3 | 77.1 | 0.0428 | 0.487 | 6.72 | 38.3 | 150.1 | 0.0012 | 0.14 | 0.878 | 3.27 | 27.3 | Avrami |
| 81 | 50 | 0.30 | 20 | 84.1 | 0.1453 | 0.796 | 5.61 | 35.0 | 85.1 | 0.0261 | 0.884 | 4.23 | 30.0 | 91.1 | 0.1406 | 0.41 | 0.979 | 1.81 | 16.7 | Avrami |
| 82 | 50 | 1.00 | 20 | 95.1 | 0.0791 | 0.959 | 4.97 | 32.9 | 96.1 | 0.0146 | 0.892 | 8.08 | 41.6 | 94.1 | 0.0863 | 1.57 | 0.982 | 3.31 | 27.6 | Avrami |
| 83 | 50 | 0.30 | 100 | 40.1 | 0.4746 | 0.124 | 3.04 | 24.0 | 40.1 | 0.1157 | 0.120 | 3.05 | 24.1 | 41.1 | 4.2209 | 0.26 | 0.183 | 2.94 | 25.4 | PFO |
| 84 | 50 | 1.00 | 100 | 60.1 | 0.2640 | 0.492 | 4.40 | 30.7 | 60.1 | 0.0520 | 0.524 | 4.25 | 30.1 | 61.1 | 0.3759 | 0.53 | 0.613 | 3.84 | 30.2 | SCD |
| 85 | 50 | 0.65 | 60 | 94.1 | 0.1525 | 0.793 | 6.11 | 36.6 | 96.1 | 0.0275 | 0.883 | 4.60 | 31.5 | 100.1 | 0.1590 | 0.43 | 0.983 | 1.77 | 16.3 | Avrami |
| 86 | 50 | 0.65 | 60 | 92.1 | 0.3463 | 0.338 | 5.96 | 36.1 | 93.1 | 0.0662 | 0.334 | 5.98 | 36.2 | 94.1 | 0.9579 | 0.36 | 0.522 | 5.06 | 35.2 | Avrami |
| 87 | 50 | 0.65 | 60 | 91.1 | 0.0903 | 0.722 | 11.28 | 47.6 | 91.1 | 0.0170 | 0.748 | 10.74 | 46.7 | 91.1 | 0.0905 | 0.81 | 0.732 | 11.06 | 49.3 | SCD |
| 88 | 70 | 0.30 | 60 | 87.1 | 9.0753 | 0.000 | 7.62 | 40.6 | 87.1 | 0.2339 | 0.000 | 7.62 | 40.6 | 150.1 | 0.0011 | 0.05 | 0.173 | 6.93 | 40.8 | PFO |
| 89 | 70 | 1.00 | 60 | 94.1 | 0.2885 | 0.712 | 3.92 | 28.6 | 95.1 | 0.0582 | 0.716 | 3.89 | 28.5 | 96.1 | 0.4964 | 0.45 | 0.913 | 2.15 | 19.8 | Avrami |
| 90 | 70 | 0.65 | 20 | 84.1 | 0.2784 | 0.547 | 4.98 | 32.9 | 85.1 | 0.0510 | 0.565 | 4.88 | 32.5 | 86.1 | 0.4701 | 0.42 | 0.820 | 3.14 | 26.6 | Avrami |
| 91 | 70 | 0.65 | 100 | 66.1 | 0.2493 | 0.568 | 4.51 | 31.1 | 66.1 | 0.0492 | 0.598 | 4.35 | 30.5 | 85.1 | 0.1308 | 0.18 | 0.875 | 2.42 | 21.9 | Avrami |
| Mean ± SD | | | |  |  | 0.592 ± 0.271 |  |  |  |  | 0.616 ± 0.281 |  |  |  |  | 0.47 ± 0.35 | 0.763 ± 0.264 |  |  |  |
| Good fits (R² > 0.7) | | | |  |  | 7/15 (47%) |  |  |  |  | 7/15 (47%) |  |  |  |  |  | 11/15 (73%) |  |  |  |
| Best model (by AIC) | | | |  |  | 2/15 |  |  |  |  | 3/15 |  |  |  |  |  | 10/15 |  |  |  |

*Table S5. Individual Avrami fits (S/L = 100 excluded)*

| **Run** | **T (°C)** | **S/L** | **k (1/min)** | ***n*** | **R²** | **Note** |
| --- | --- | --- | --- | --- | --- | --- |
| 1 | 35 | 60 | 0.3079 | 0.200 | 0.889 |  |
| 2 | 35 | 20 | 0.1654 | 0.607 | 0.977 |  |
| 3 | 35 | 60 | 0.3011 | 0.626 | 0.919 |  |
| 4 | 50 | 20 | 0.1406 | 0.410 | 0.979 |  |
| 5 | 50 | 20 | 0.0863 | 1.570 | 0.982 |  |
| 8 | 50 | 60 | 0.1590 | 0.431 | 0.983 |  |
| 9 | 50 | 60 | 0.9579 | 0.359 | 0.522 |  |
| 10 | 50 | 60 | 0.0905 | 0.811 | 0.732 |  |
| 11 | 70 | 60 | 0.3161 | 0.400 | 0.725 | t=5 excluded |
| 12 | 70 | 60 | 0.4964 | 0.450 | 0.913 |  |
| 13 | 70 | 20 | 0.4701 | 0.421 | 0.820 |  |

Figure S1. XRD patterns of the EoL LFP-based battery black mass (red) and precipitate after oxalic acid leaching (blue), compared with the LiFePO₄ reference pattern (PDF 98-018-9057, bottom). Square markers (■) indicate LiFePO₄ reflections, while triangle markers (▲) show graphite.

Figure S2. EDS of industrial black mass

## *Figure S3. Grey image of EoL LFP-based black mass, overlay and individual metals from EDS (O, C, Al, Fe, P and Cu). Magnification 1000x, acceleration voltage 15 kV. 3.2 Box-Behnken design and model fitting*

Figure S4. Extraction of Li, Al, P, Fe and Cu as a function of time and grouped by temperature, acid concentration and S/L ratio. Mean data for all experiments shown with standard deviation as error bars.

Figure S5. Predicted Li extraction from RSM model vs observed Li extraction

Figure S6. Evolution of the reduced RSM response surface (Acid concentration × S/L ratio) over leaching time. The four-term model (Y = β₀ + β₂x₂ + β₃x₃ + β₂₂x₂² + β₃₃x₃²) was fitted independently at each time point. Optimal conditions (★) are shown only for time points where the reduced model achieved adequate fit (R² ≥ 0.70).
